# “I’m a bit middle class, a bit working class, a bit white and a bit Caribbean” - the retention of nurses in general practice and the intersection of professional and societal level cultural and structural issues: a qualitative interview study

**DOI:** 10.1186/s12913-025-13420-2

**Published:** 2025-10-09

**Authors:** Helen Anderson, Louise Brady, Joy Adamson

**Affiliations:** 1 York Trials Unit, Department of Health Sciences, University of York, York, YO10 5DD UK; 2 Selby, North Yorkshire, UK

**Keywords:** Primary care, General practice nursing, Culture, Structural issues, Gender, Ageism, Social class, Ethnicity, Race, Medical hegemony, Professional identity

## Abstract

**Background:**

The global nursing workforce crisis has worsened since the Covid-19 pandemic. In England 10% of nursing posts are unfilled, with the general practice nursing workforce being particularly vulnerable across England and Wales. Similar challenges are reflected internationally and pose a risk to patient care and the long-term future of general practice. However, primary care nursing is under-researched and factors which support or challenge retention of nurses in general practice are poorly understood. In this paper we aim to explore the influence of the intersection of cultural and structural factors underpinning retention of nurses in general practice.

**Methods:**

An exploratory qualitative interview study was conducted. Professional and social media networks and snowballing techniques were used to recruit. Forty-one participants were interviewed who were either working in, or who had worked in, nursing teams in general practice across England and Wales, as well as nurse leaders. Data were collected between October 2023-June 2024. Framework Analysis was used with Bourdieu’s concept of Capital used as a sensitising concept. University of York ethics approval (Ref: HSRGC/2023/586/A) was gained and the study was funded by the General Nursing Council Trust.

**Results:**

Professional and societal level constructs influenced and impacted on cultural and structural issues associated with retention of nurses working in general practice in our study. Gender, social class, race, ethnicity and age intersected with each other, alongside medical hegemony and professional identity to shape nursing in general practice. Analysis indicates nurses in general practice lack, or - just as importantly - are perceived to lack, social, economic and cultural capital and this impacts on their position within general practice, results in perpetuation of social disadvantage, reproduces inequality and contributes to the devaluing of nursing, ultimately contributing to attrition.

**Conclusions:**

The intersection of the underpinning cultural and structural factors identified indicate why retention of nurses in general practice is difficult to resolve. We suggest that the first steps are to raise these factors to a conscious level and argue that unless they are acknowledged, and work to address them is undertaken, strategies to support retention of nurses working in general practice may be unsuccessful.

**Trial registration:**

Registered at Open Science Framework: https://osf.io/2byxc/.

**Supplementary Information:**

The online version contains supplementary material available at 10.1186/s12913-025-13420-2.

## Background

The value of nursing is ‘almost inestimable’ and the backbone of healthcare worldwide [1]. However, there is a global nursing workforce crisis which has worsened since the Covid-19 pandemic. The International Council of Nurses estimate an increased shortfall in nurses globally from 6 million pre-pandemic to up to 13 million due to increased need, an ageing workforce and poor retention [2]. Attrition negatively impacts on patient care and safety [3]. In the United Kingdom, Brexit (withdrawal from the European Union) has also depleted the nursing workforce with negative consequences [4]. In England 10% of nursing posts are unfilled [5], with the general practice nursing workforce being particularly vulnerable. It is estimated that 25–50% general practice nursing posts in England may be unoccupied within less than 10 years [6]. Similarly, the general practice nursing workforce in Wales is increasingly ageing [7], with similar challenges to the primary care nursing workforce reflected internationally [8, 9]. As nurses are core to general practice [10], these shortages are highly concerning as they pose a risk to patient care and the long-term future of general practice [6, 7]. However, primary care nursing is under-researched [1] and there is a paucity of research focusing on the factors which support or challenge retention of nurses in general practice, with most research concentrating on secondary care roles and failing to consider the specific context and experiences of primary care nurses [11]. While for healthcare workforces more widely, focus is on recruitment rather than retention [12]. Consequently, we carried out a study exploring the retention of nurses in general practice [13] which identified cultural and structural issues underpinning retention of nurses. In our current paper we aim to analytically explore these cultural and structural underpinnings associated with retention of nurses working in general practice and offer insight into how this work can be harnessed to address retention moving forward.

## Theoretical framework

The study is situated against the backdrop that, as a profession, nursing is underappreciated despite its central role in strengthening primary care [1]. It has been postulated that nursing lacks appreciation, respect and is not appropriately valued due to unconscious bias related to its nearness to fundamental care delivery and the cumulative effects of gender, ethnicity and age [14]. Similarly, cultural forces intersect to produce prejudice and discrimination underpinned by status differentials between medicine, managerialism and nursing [15, 16]. These contribute to a lack of involvement of nurses in decision-making, which has been implicated in retention [17, 18]. When the value of a professional group is diminished, retention is negatively affected [12].

In this paper we draw on Bourdieu’s concept of ‘capital’ as a sensitising concept within which to frame data analysis. Sensitising concepts are central organising ideas which can be developed from existing theory to make sense of a data set. The researcher ‘develops repackages and refines’ an idea or concept to support sense-making and move analysis from descriptive to interpretive [19].

For Bourdieu, society is not a level playing field and the concept of capital can explain how inequalities occur [20]. The structure of the distribution of capital reflects how the social world is organised and functions, with the life chances of different players being unequal. Capital is often ‘inherited’ through life experiences and upbringing [20]. Bourdieu regards capital as both economic and symbolic and it develops, in part, from social class structures [21]. Economic capital relates to wealth, income and financial assets, while symbolic capital encompasses cultural and social capital. Cultural capital is the symbolic representation of an individual’s socio-economic status, such as level of education (and level of respect for that education), access to culture and cultural objects such as books or art, and knowledge of what is culturally acceptable in specific environments. Social capital refers to contacts, connections and social networks [22]. Capital is posited as one of the ways in which power and authority is subtly and invisibly gained and maintained by individuals and groups [21]. While economic capital (or lack of it) is a well-recognised contributor to unequal power structures, cultural and symbolic capital are considered to be more hidden forms [20] and consequently their role in multiprofessional fields may be overlooked.

A key underpinning aspect of Bourdieu’s understanding of capital is that of competition between individuals and groups, with different forms of capital being used or exchanged to gain or keep power [21]. However, gender and class often intersect to confer a ‘double disadvantage’ for working class women [22]. While race [23, 24], ethnicity [14], and ageism [25] can also intersect to negatively impact on the nursing workforce. We draw on Bourdieu’s understanding of different forms of capital as a sensitising concept and explanatory framework within which to understand how, why, and in what ways, culture and structure intersect and impact on retention of nurses in general practice.

## Methods

### Aim

To understand the influence of the intersection of cultural and structural underpinnings on retention of nurses in general practice.

### Objectives

To explore the intersection of cultural and structural factors associated with retention.

To identify how, why and in what ways, general practice culture and structure influences, and is associated with, retention.

To raise cultural and structural issues to a conscious level which may support employers and policy makers in future primary care workforce planning, and to support individual nurses in negotiating and making decisions about their work, within the wider cultural and structural context.

### Study design and setting

We carried out a qualitative interview study [26] with members of general practice nursing teams across England and Wales and local and national nurse leaders. Using a social constructivist approach, and drawing on Bourdieu’s concept of Capital [20, 21], we considered how factors influencing retention were underpinned by structural and cultural issues. The study was funded by the General Nursing Council Trust [Ref: 2023-09, approved July 2023] and was conducted over a 12-month period from 1 September 2023 with interview data collected between October 2023-June 2024.

### Participants

We interviewed members of nursing teams working in general practice in England and Wales at different professional levels ranging from general practice nurses, health care assistants, advanced nurse practitioners, trainee advanced nurse practitioners, nursing associates, assistant practitioners and nurses in management/leadership positions. Those who had recently left general practice, either to move to other roles or to retire, were eligible to take part. We also interviewed local and national leaders.

### Recruitment

Professional and social media networks were used to engage recruitment [13, 27]. Study details were posted on X (previously Twitter) by the lead researcher [HA] and nursing groups such as @WeGPNs, @RCNGPNForum, @BAMEGPNs and @TheQNI, were asked to disseminate study information with the aim of achieving maximum and diverse variation of representation. The study team also used national and targeted professional networks to invite specific groups. For example, to support engagement with global majority participants, nurse leaders advised and facilitated promoting the study in specific geographical areas. Details about the study were also published by a professional nursing journal, leading to additional recruitment.

### Sampling strategy

It was estimated that approximately 30–40 interviews would provide appropriate depth and breadth of data [28] and a phased recruitment process was followed. Initially, we used convenience sampling to recruit opportunistically and through this we collected demographic participant data on key personal and workplace characteristics [additional file 1]. Following this, specific groups were purposively targeted based on recruitment gaps. For example, health care assistants did not initially respond to promotion of the study, so snowball sampling was successfully used to engage with this sub-group, with recruited participants passing on study details to their health care assistant colleagues. An information sheet was provided and written informed consent was obtained from participants, who were thanked for giving up their time with a £50 gift voucher.

### Data generation

The lead researcher [HA] carried out semi-structured interviews via an online platform or telephone at a time and date suitable for participants. Interviews ranged between 45 and 90 min and were audio-recorded. They were informed by an iterative topic guide [additional file 2] developed from existing literature and study aims and objectives [29]. A professional transcription service transcribed interviews verbatim. Following this, audio files were deleted.

### Data analysis

A framework approach [29] was taken. This involved systematic analysis by the lead researcher [HA] which was both grounded in the raw data and drew on concepts developed from the research question/objectives and wider literature with Bourdieu’s concept of Capital [17] used as a sensitising concept. That is, after familiarising ourselves with the data and developing the more descriptive themes set out in our main findings paper [13], the research team drew on our wider knowledge and understanding of broader literature, from which it became intuitively clear that Bourdieu’s concept of Capital resonated with participants’ explicit and implicit narratives. After further reading and discussion with a sociologist colleague we analysed the data according to how this related to Bourdieu’s concept of Capital and interrogated the data for deeper meaning [19]. This involved mentally moving backwards and forwards between conceptual ideas and the empirical data and allowed themes to be developed at a conceptual, rather than descriptive level [19]. We also searched the data set to identify negative cases and alternative explanations. Framework analysis [29] follows the steps of: data familiarization; thematic framework construction; data labelling and sorting (coding); review of data extracts; summarising and presenting (charting) data; abstraction and interpretation. This enabled testing of potential themes and relationships in the data. However, while this describes the procedural steps taken in our analysis, it does not highlight what Van Maanen [30] describes as the ‘headwork’ required to make sense of the data at an interpretive level where the researcher’s wider reading, previous work, understanding of the broader field and underpinning experiences help shape the analysis. Because of this, interpretive analysis relies on an element of trust, rather than following positivist ideas of reproducibility [19]. As analysis was undertaken manually, no data management software was used.

### Ethical considerations

Research governance approval was gained from University of York Research Governance Committee (Ref: HSRGC/2023/586/A) on 29th September 2023 and the study was carried out according to the Declaration of Helsinki. General Data Protection Regulations, the Data Protection Act (2018) and the University of York’s Data Management Policy were followed. Unique identifying numbers were used to pseudonymize identifiable data. The relatively small number of participants and detailed information generated could lead to the potential for participant identification. This was taken into consideration and addressed when presenting information in this paper.

### Rigour and reflexivity

Holloway’s [31] conception of qualitative rigour underpinned the study in that we developed thick description and compared and linked findings to theory and prior work. We aimed for our findings to resonate with experiential knowledge [31] and reflect shared experience [32] in order to achieve naturalistic generalizability [32]. Reporting is per COREQ [33].

Two study team members [HA and LB] are registered nurses with general practice experience, and one is a non-clinical methodologist [JA]. All team members are White, female and would consider ourselves to be from working class backgrounds. As this has the capacity to impact on the research, we took a reflexive approach throughout the study and during regular research team meetings. Through this the team continuously questioned analytical thoughts and sought alternative explanations.

## Results

### Characteristics of participants

Forty-one participants across England and Wales (England *n* = 35; Wales *n* = 6) took part and their details are described in Table 1. We interviewed a range of nursing team members from newly qualified to those who had retired, including general practice nurses, health care assistants, advanced nurse practitioners, nursing associates and assistant practitioners, alongside local and national nurse leaders. The majority were currently working in general practice while some had already left, with others considering leaving or actively looking for alternative employment. Participants had earned qualifications up to and including post graduate level and many held prescribing qualifications. Seventeen of the participants were remunerated at equivalent of Agenda for Change Band 7 or above. This was because participants included national and local nurse leaders and those working at an advanced scope of practice (e.g. advanced nurse practitioners, independent prescribers). Participants’ workplaces varied in terms of practice size, region, rurality and deprivation scale (Table 2). A joint interview was carried out with two non-nurses, recruited through snowball sampling, because they were working to support retention of nurses in general practice in a specific area of England and were identified as key informants. Most participants were female (*n* = 35 female; *n* = 6 male) and thirty-two self-identified as white.

**Table 1:** Participant characteristics

Participant Demographic Data *N* (%)	
Gender	
Female	35 (85.4)
Male	6 (14.6)
Country of Work	
England	35 (85.4)
Wales	6 (14.6)
Ethnicity (self-defined)	
White British	25 (61)
White Irish	2 (4.9)
White Northern Irish	1 (2.4)
White European	1 (2.4)
White Welsh	3 (7.3)
Black British	1 (2.4)
White Indo-Caribbean	1 (2.4)
Black	1 (2.4)
British Indian	2 (4.9)
British Asian	1 (2.4)
Mixed	1 (2.4)
Black African	1 (2.4)
Mixed White	1 (2.4)
Age Range	
20–29	4 (9.8)
30–39	8 (19.5)
40–49	12 (29.3)
50–59	12 (29.3)
60–70	5 (12.2)
Role	
General Practice Nurse	10 (24.4)
Advanced Nurse Practitioner	6 (14.6)
Registered Nursing Associate/Assistant Practitioner	4 (9.8)
Health Care assistant	3 (7.3)
Trainee Advanced Nurse Practitioner (currently General Practice Nurse)	4 (9.8)
General Practice Nurse/Advanced Nurse Practitioner combined role	3 (7.3)
Nurse Leader	7 (17.1)
Other primary care role	1 (2.4)
N/A	2 (4.9)
Qualifications	
Registered Nurse (RGN)	7 (17.1)
RN (Dip) Adult	5 (12.2)
RN (BSc)	16 (39)
RN (RSCN)	1 (2.4)
RN (MSc/PGDip)	2 (4.9)
RN(not stated)	4 (9.8)
ANP MSc/PGDip	9 (22)
Non-Medical Prescriber	10 (24.4)
Foundation Degree (Nursing Associate/Assistant Practitioner)	4 (9.8)
Health Care assistant level 3/4	3 (7.3)
Other MSc/MA	7 (17.1)
Other Batchelor’s degree	4 (9.8)
Other post grad quals	9 (22)
N/A (not a nurse)	2 (4.9)
Years Qualified	
1–9	6 (14.6)
10–19	6 (14.6)
20–29	5 (12.2)
30–39	9 (21.2)
40–49	4 (9.8)
Not stated	2 (4.9)
Registered Nursing Associate/ Assistant Practitioner	1-9yrs: 3 (7.3) 10–20 yrs: 1 (2.4)
Health Care Assistant	1–9 years: 3 (7.3)
N/A (not a nurse)	2 (4.9)
Employment Status	
In general practice	23 (56.9)
Left general practice	3 (7.3)
Retired (early)	1 (2.4)
Retired early and returned (different role)	3 (7.3)
Left for another role in primary care	2 (4.9)
Nurse Leader	7 (17.1)
Not applicable	2 (4.9)
Pay per hour/Agenda for Change equivalent (2024)	
Band 3 (£11.67-£12.45/hour)	2 (4.9)
Band 4 (£12.86 -£14.21)	6 (14.6)
Band 5 (£14.53-£17.69)	2 (4.9)
Band 6 (£18.10–21.80)	6 (14.6)
Band 7 (£22.37-£25.60)	9 (22)
Band 8a (£26.06-£29.33)	5 (12.2)
Band 8b (£30.16-£35.04)	1 (2.4)
Band 8c (£36.01–41.50)	1 (2.4)
Band 8 d (£42.74-£49.29)	1 (2.4)
Band 9	2 (4.9) (nurse leaders)
N/A	5 (12.2)
Not stated	1 (2.4)

**Table 2:** Practice descriptors

Practice Descriptors (32 practices)	
Small (<10k)	14 (43.8)
Medium (10-20k)	15 (46.9)
Large (>21k)	2 (6.3)
No info	1 (3.1)
N/A	9
Region	
England (north west)	6 (18.8)
England (Yorkshire & north east)	4 (12.5)
England (midlands)	4 (12.5)
England (south east) including London	12 (37.5)
England (south west)	0 (0)
Wales	5 (15.6)
No info	1 (3.1)
N/A	9
Rurality	
Rural/semi-rural	6 (18.8)
Suburban	5 (15.6)
Urban	16 (50)
Mixed	3 (9.4)
Other	1 (3.1)
No info	1 (3.1)
N/A	9
Practice Population Deprivation Level*	
1 (most deprived)	2 (6.3)
2	5 (15.6)
3	6 (18.8)
4	3 (9.4)
5	0 (0)
6	3 (9.4)
7	0 (0)
8	4 (12.5)
9	4 (12.5)
10 (least deprived)	1 (3.1)
No data	3 (9.4)
N/A	10
All practices (where applicable) were CQC rated as good except: 1 -requires improvement & 1 - rated outstanding	

*National General Practice Profile data (https://fingertips.phe.org.uk/profile/general-practice) or https://statswales.gov.wales/Catalogue/Health-and-Social-Care/General-Medical-Services/General-practice-population

### Overview

This paper discusses professional and societal level constructs which, both directly and indirectly, influenced and impacted on cultural and structural issues associated with retention of nurses working in general practice in our study. Gender, social class, race, ethnicity and age intersected with each other, alongside medical hegemony, healthcare hierarchy and professional identity to shape how nursing in general practice was perceived and enacted. The social, historical and political context within which nursing, and nursing in general practice within that, is situated, as well as the nuanced experience of modern-day nursing in general practice, impacted on retention in a number of ways. Drawing on Bourdieu’s concept of Capital [20, 21], our analysis indicates that nurses in general practice lack, or - just as importantly - are perceived to lack, the social, economic and cultural capital required to exchange or bargain, and this impacts on their position with general practice, particularly in relation to their general practitioner employers. This results in the perpetuation of social disadvantage and reproduces inequality and the devaluing of nursing. How this shapes what nursing looks like in general practice, and how this ultimately impacts on retention of this highly skilled group, is explored below.

Because the study includes members of general practice nursing teams at different levels, ‘nurse’ or ‘participant’ refers to members of the nursing team to aid readability, unless stated otherwise, when terms such as ‘registered nurse’, ‘general practice nurse’, ‘advanced nurse practitioner’, ‘nursing associate’ or ‘healthcare assistant’ are used as appropriate. While we aimed to use a ‘spread’ of participant quotations to support our findings in this paper, and link these directly to the raw data, we have followed Sheard’s [19] lead of using verbatim quotations which best articulate our interpretations. Consequently, some participants are quoted more than once and, because of the direction of this paper, there is a tilt towards those in leadership and more senior nursing positions.

### Gender ‘pink collar’ work and the historical and socio-political relationship between nursing and medicine.

The general practice nursing population in terms of gender, age and ethnicity [as per NHS England data: Additional File 3] was broadly reflected in our study sample at the time of data collection, although males were slightly overrepresented. Nursing is a predominantly [89%] [34] female profession. However, nursing in general practice is further skewed with only 1.7% of general practice nurses being male, although this increases to between 6% and 9.1% for males working in specialist and advanced practice roles [Additional File 3]. In our study, this resulted in an emphasis on nursing in general practice being seen as *women’s work* and consequently not valued because such ‘pink collar work’ is highly gender-segregated and poorly paid [35]. Participants in our study reported being likely to work in general practice, at least in part, to manage caring and family responsibilities, which limited their freedom of labour and reduced their economic capital. Furthermore, much of general practice nursing work could be understood as gendered in terms of provision of women’s health, cervical cytology, childhood immunisations and what could be seen as ‘lower status’ non-acute, ‘softer skilled’ work. This meant that nursing in general practice lacked symbolic and, as a consequence, economic capital. This could be considered to contribute to the lack of recognition of the value of this highly skilled and educated, but politically weak group.

*it is women’s work and that introduces another element of control ….You’ve got women who have generally got children*,* who are needing more social hours and then accept poorer terms and conditions*,* and its near home. [It’s] about women’s situation because it is women who have to do this. Some of the practice nurse role is more female related as well. There’s a massive part of women’s health in general practice*,* you’ve got smears*,* contraception and HRT [hormone replacement therapy]*. *[ID 122*,* Advanced Nurse Practitioner*,* Female].*

Poor maternity pay and leave entitlement was considered a major negative factor in retaining a largely female workforce, although it had a negative impact on male participants as well, *‘Maternity pay is not there at all [which is a] major barrier for women who are wanting a family….It definitely worries me because I’ve only got two weeks [paternity leave]’ [ID 132 General Practice Nurses/trainee Advanced Nurse Practitioner*,* Male].* More broadly, balancing tensions between parenthood and career, combined with wider societal pressures, also contributed external stresses which were considered to disproportionally affect women.

*When I look at how people around me have developed in their career and*,* particularly the men*,* how they are more senior than me? And the gender pay gap is a real thing. You know those compromises that you make?….If you’ve got children*,* you feel guilty and almost like a sinner*,* the mum guilt is a real thing*,* and you feel like you’re failing at everything. [ID 129 Regional Nurse Leader, Female]*

The gendered nature of nursing work in general practice was also related to nurse’s lack of strategic and decision-making input. This was highlighted by some participants as negatively affecting retention and linked with the established historical and socio-political relationship between nursing and medicine. Roles considered to be closer to medicine, such as advanced nurse practitioners, drew greater economic and symbolic capital than other nursing roles. Consequently, the lack of value for work which was seen to align more closely to women’s work was reflected in poorer pay, terms and conditions.

*as soon as somebody becomes an ANP [advanced nurse practitioner] they take them out [of nursing]. The ANP [advanced nurse practitioner] sits in a room next to the doctor. They’re not in uniform*,* and they totally almost put them on the pedestal…the ANP [advanced nurse practitioner] gets a Band 8A or 8B. [ID 120*,* General Practice Nurse*,* Female]*.

That the nursing profession is perceived to be gendered female [36] reflected how nurses were expected to behave, in comparison to their medical counterparts, for the nursing voice to be heard. Reminiscent of Stein’s (1968) *doctor-nurse game* [37], some participants recognised the importance of offering challenge in ways which did not destabilise the established healthcare hierarchy. Not *playing the game* could have potential negative consequences.

*when I first started at the CCG [Clinical Commissioning Group]*,* my manager said*,* ‘you can’t be a hot head. GPs [general practitioners] can*,* but you can’t’*,* and then I realised it was much bigger. When you get invited to a meeting…You’re there to keep your mouth shut*,* so you’ll be invited next time.…It’s more of a cultural thing*,* it’s very hierarchical*,* they’ve not moved with the times and so now [nurses] need to really stand up and use their skills properly and not in a shouty way. It’s that quiet leadership that we don’t talk about. You need to be able to make sure that it’s their idea. [ID 205, National Nurse Leader, Female]*

### The intersection of class, gender, medical hegemony, professional identity and healthcare hierarchy

Some participants indicated that social class, or perceptions of this, influenced nurses’ position and recognition in general practice and was reflective of society more broadly.

This intersected with gender to impact on the professional status of nursing in general practice. Participants recognised that nurses needed to be formally supported to develop skills to fully take opportunities available to contribute at higher levels of decision-making and professional discourse.

*[medicine] has the persona of men*,* so you get this dominant effect*,* this ‘we know best’. When I’m in meetings I hear GPs [general practitioners] speaking with real authority. Nurses are almost apologetic for their view. I’m a nurse*,* I’m a man*,* there are moments when I even hear myself [with] that imposter syndrome…. Some of that is probably tied to issues around class. There is something around your background and education*,* your thinking….If you don’t come from that background*,* you don’t feel you should be there….If we’re thinking about retention of nurses*,* we need to offer more leadership development*,* support around issues with self-confidence. [ID 203, National Nurse Leader, Male]*

However, the practicalities of attaining decision-making positions were dependent, to some extent, on opportunities related to social class and the economic capital to invest in general practice as a business. This was also inhibited by nurses being seen, by themselves and others, as ‘doers’ rather than leaders, with nurses lacking the symbolic capital to aim for, and be accepted as, professionals who can make a contribution to healthcare leadership.

*Unless you’re married to a GP [general practitioner]*,* which still happens a lot*,* nurses are still seen as*,* ‘go and sort the patients out*,* I’ll [the general practitioner will] lead*,* I’ll manage’. Also*,* traditionally nurses don’t have the money to invest in [general] practices….It’s almost down to class. Doctors are normally a bit middle/upper class traditionally and they’ve money to invest…. I think some nurses don’t want to either. Again*,* back to education because we’re told*,* ‘oh you’re just a nurse*,* just aim for your level and don’t aim for the sky’. So*,* some nurses have stayed in their lane. [ID 102, General Practice Nurse/trainee Advanced Nurse Practitioner, Male]*

That there was a class distinction, and its impact on relationships between doctors and nurses, was considered by some to be so embedded that it was unconscious and unintentional and thought to play out in the actions of both professional groups. This meant that even when nurses did have a seat at the decision-making table, disparity was felt in decision-making and beyond. One nurse participant, who was a practice partner, felt that the difference in social class between herself and her general practitioner colleagues affected how she perceived herself and how she was perceived by others.

*The thing that jumped out at me was the class thing. I feel quite naive at the table because*,* conversely to my GP [general practitioner] colleagues*,* who have all had a certain level of education and have all come from very similar backgrounds*,* I grew up on a council estate….my accent is very working-class and very different from everybody else in the partnership. I don’t know that I’m treated differently*,* but I know I feel different. I don’t know whether that’s because there are some unconscious microaggressions or whether that is just me feeling on the back foot. [ID 126, General Practice Nurse and Nurse Partner, Female]*

For this nurse in a senior position, wider cultural, social and economic disparity meant she did not always feel equal to others and assumptions were made on both parts. She was not remunerated at the same level as her general practitioner partner colleagues because perceptions of her nursing contributions were that they were *less than* her medical colleagues. Consequently, even when invited to contribute, it was unlikely nurses would have the economic, social or cultural capital to level the playing field. Furthermore, the accepted legitimacy that medicine’s contribution was greater than that of nursing meant that the social and economic mobility (and capital) of nurses was stymied.

*My interests are very different from the rest of the partnership and that feels quite stark in stereotypical areas that you would associate with class. Around music and activities and having disposable income. I’m not in that same position…. The parity in terms of the partnership is different because I’m not a doctor. So*,* it means that my salary*,* my drawings are not the same as the medical partners but there is parity in terms of decision-making and profit percentage which I agreed felt fair….But equally that’s still inequity*,* the point is*,* that bit’s not going to ever change*. *[ID 126, General Practice Nurse and Nurse Partner, Female]*

Related to this, the perception of nursing and medicine within a traditional professional hierarchy was seen as inculcated within both professions and contributed to the roles and expectations of each professional group, *‘there’s that traditional model where I’m the doctor*,* you’re the nurse*,* you’re somewhere below me’ [ID108*,* General Practice Nurse*,* Female].* This was considered to be perpetuated by the historical and socio-political context within which both nursing and medicine operate, and the medical hegemony and healthcare hierarchy associated with this. For participants who could be perceived as *lower down* in a traditional healthcare hierarchy, they sometimes found themselves excluded from valuable support. This led to a perceived disconnect between, and within, professional groups and a feeling of *them and us*, which participants recognised was detrimental to effective working relationships and risked demoralising some team members as well as negatively impacting on retention.

*There’s a bit of hierarchy. Every morning there is a huddle to look at the triage list. Health care assistants and practice nurses [a]re not invited. But other stuff happens at that meeting that you miss out on*,* that team building aspect to that huddle. That’s quite beneficial for them and the rest of us [are] lacking that. It subconsciously drives a bit of an “us and them” thing….It has been raised a couple of times that we miss out*,* but it’s not changed. [ID 118, Health Care Assistant/Nursing Student, Female]*

The relative positions of medicine and nursing within the established healthcare hierarchy led to a perception of ownership and control over nurses, rather than nurses being seen as equal and able to offer valuable, but different, perspectives.

*I was invited to a GP [general practitioner] locality meeting. It was all GPs [general practitioners] and practice managers*,* and I said*,* ‘I can’t see any nurses here’. The practice manager said*,* ‘I don’t pay my nurses to come to meetings*,* I pay them to see patients’. I was absolutely speechless….I’ve never forgotten that experience. That ownership of [nurses]. That’s a cultural thing as well*,* ‘my nurses belong to me’. That plays into a culture that nurses are seen as being owned by the practice rather than an equal team member that has a different perspective to bring. [ID 201, National Nurse Leader, Female]*

Even when nurses were ostensibly invited to meetings or pushed themselves to be heard at a more strategic level, they were sometimes shut down and their contribution discounted because of the status, and lack of symbolic capital, of nursing.

*They were having a PCN [Primary Care Network] meeting. It was an open invite*,* so I decided I would go. I was the only nurse there*,* and I was ignored for at least 15 min when they were doing introductions by the GP [general practitioner] who was running it. I had to be very dominant to get myself noticed and if I’d been a lesser character I would have probably just left because it was quite awkward. The Chairman of the meeting [introducing another newcomer] said*,* ‘I’d like to introduce somebody new*,* this is Dr [name]’ and the GPs [general practitioners] were like*,* ‘welcome*,* nice to see you’. Then he just started the meeting. I put in the chat box*,* ‘Hi*,* I’ve joined the meeting too as a new person and I’m hoping to give a nursing perspective’ and it was completely ignored! After the meeting I sent an email to say I’d be interested in giving a nursing perspective and I didn’t even get a response….It’s because I was a nurse and that’s an example of what you’re up against. [ID122, Advanced Nurse Practitioner, Female]*

Linked to this, participants often saw their professional nursing identity in terms of putting the patient, and profession, first. They experienced dissonance between this and asserting their own value. Participants often stressed that as nurses, remuneration was not their first priority, but rather they work in general practice for the benefit of patients and felt that negotiating for more money, or better employment terms and conditions, was contrary to their professional nursing identity.

*money has never been a driver for me as a nurse. As long as I can live to my means*,* it’s never really been a thing.….I was bending over backward for patients. I would always do that. I’m not money driven but there came a point where I was a bit fed up….it didn’t bother me until I started to feel like it was being taken advantage of*. *[ID 117, Ex General Practice Nurse, Female]*

However, due to the nature of their employment, nurses in general practice were required to individually negotiate terms and conditions directly with general practitioner employers and this was challenging within the constraints of medical and managerial hegemony. Consequently, nurses often lacked the symbolic capital to negotiate to strengthen their economic capital, and this had a negative effect on retention, *‘it’s staggering the way you get treated in primary care….we wonder why retention is poor….But if staff aren’t treated as well as they should be. So*,* yeah*,* they dropped my salary by £X an hour’. [ID 111*,* General Practice Nurse Retired and Returned*,* Female]*

The economic capital of registered nurses could also be seen to be driven down by the introduction of apprenticeship schemes and the development of nursing support roles. This negatively affected not only retention, but recruitment, in that roles previously undertaken by registered nurses were in some cases being replaced by cheaper nursing associates and health care assistants. From a positive viewpoint, these schemes could be seen to widen access to the profession in terms of social class, academic attainment and helping those with additional responsibilities into the nursing workforce, *‘I didn’t have my GCSE from school in maths and English. So*,* they paid for it. I did my GCSE evening class’ [ID 125*,* Assistant Practitioner*,* Female].* However, such policies reduced the professional status, and therefore symbolic capital, of nursing through the expansion of support roles. Participants considered that those making decisions about nursing work did not necessarily understand the importance of registered nurses in delivering and overseeing patient care, thus potentially negatively impacting patient safety, quality of care and risking exploitation. It also meant that registered nurses had less control over their employment, their professional status and, in some circumstances, their economic capital was reduced.

*Nursing associates [carrying out cervical cytology]. Oh! Who thought that was a good idea? As a GPN [general practice nurse] …It’s having that advanced understanding. Underpinning knowledge*,* skills and competence holistically that provides the care for that person. Not a task orientated process which is what’s happening now…. It’s about money and that’s the problem….it’s a concern*,* with all due respect to practice managers and GPs [general practitioners]*,* they don’t understand the concept that a nursing associate [or] a HCA [health care assistant]*,* has to work under [the supervision of] a registered nurse. That’s where it starts to get muddled*. *[ID 206, National Nurse Leader, Female]*

Participants felt that nursing’s professional associations did not hold the same social, cultural and economic capital as their medical counterparts, and this led to them not always feeling supported in relation to negotiation of pay and conditions, ‘*GPs [general practitioners] have it in their BMA [British Medical Association] specified contract that they will get these pay lifts because the BMA [British Medical Association] fight for them….Where is our support? (ID 102*,*General Practice Nurse/trainee Advanced Nurse Practitioner*,* Male).* Furthermore, it was perceived that because of nursing’s weak professional status in relation to the dominance of medicine, there was a lack of standardisation of protections, such as human resource policies, for nurses working in general practice. In addition, some nurses were considered to experience isolation from support at a broader level, because access to this was only through general practitioners and managers who acted as gatekeepers and in this way their social capital was reduced.

*it’s difficult to get an ‘in’ [with nurses in general practice] without going through the [general] practices. We’re doing work to try and make connections*,* get those stakeholders in the practices on board and then start to hopefully find ways to link in with nurses. That would be helpful [because] that at the minute is lacking. [ID 209, Non-Nurse Local Leader, Female, joint interview]*

Participants in this study had to move beyond these challenges for themselves, and others, to be heard. When participants did persevere and participate, they were sometimes listened to, and their value became evident. However, it remained that decision-making was made predominantly by general practitioners, represented by specific general practitioner bodies, and there was no equivalent organisational structure where nursing issues, such as retention, could be addressed at a strategic level. It is apparent that nursing as a profession lacked symbolic capital and, as a result, relied on the benevolence of medicine to have a voice, but often this was deficient.

*Taking on the LMC [Local Medical Committee] role*,* I sat in this meeting and thought*,* ‘I’m the only nurse here’. It’s such a different culture. They talk about stuff which is very nurse relevant but in a very different way. You suddenly feel quite exposed*,* and I felt a bit shy speaking up*,* which I wouldn’t usually. It’s made me realise that [the Local Medical Council] is a GP [general practitioner] representative body*,* I’m a nurse observer. They seem to be listening to what I say*,* but there isn’t that voice in general practice for nurses. A lot of stuff they’re dealing with is heavily to do with nurses*,* but there’s not much nurse representation on the decision-making side. There’s also no one to say*,* ‘nurse retention is a problem*,* so let’s use this nurse representative body to fix that’. That body doesn’t seem to exist! [ID133, General Practice Nurse/trainee Advanced Nurse Practitioner and Local Medical Committee Lead Nurse, Female]*

### The intersection of culture, race, ethnicity, gender and class

Ethnicity, race and cultural background were also considered by some to intersect with gender and, particularly, social class to impact on retention of nurses in general practice. These intersections meant that the cultural capital of this group of nurses was multiply affected. Associated relationships with both patients and other clinical team members, could negatively impact on retention.

*There was a cultural thing. We’re an affluent area. Patients are older*,* middle*,* upper class and quite posh. They talk in a certain way*,* and you have to feel comfortable dealing with that. The nursing associates struggled a little bit with that….Three of them were black and one was Turkish. The one that left in the beginning found it difficult compared to working in an area where people were more like them. People do struggle with racism. I don’t think it was necessarily an overt thing*,* although one nursing associate who stayed a bit longer*,* a patient didn’t want to be seen by her*,* and she felt that was because she was black*. *[ID 133, General Practice Nurse/trainee Advanced Nurse Practitioner and Local Medical Committee Lead Nurse, Female]*

It was also highlighted that the majority of nurses working in general practice are white, with nurses from different racial or ethnic backgrounds concentrated in areas of high diversity such as London. This led to concerns that culturally appropriate nursing care, and the needs of diverse populations outside of large cities may not be fully met. It was acknowledged that societal level considerations, such as strategies to address racism, were required to support recruitment and retention of global majority nurses into general practice.

*General practice nursing is predominantly white women of a certain age. How does a practice deliver a service to a diverse population from just one group ethnicity? How do we attract more nurses from the global majority? You already have racism and lack of career progression for the global majority in the NHS [National Health Service]*,* particularly in acute and community care. What chance have we got of pulling them into general practice? It’s something we need to address*,* or we won’t hold on to that staff either*. *[ID 203, National Nurse Leader, Male]*

There was also an acknowledgment that as international nursing recruitment grows within general practice, there needs to be consideration of the intersection between culture and gender, and how nurses can be supported, ‘*females are very quiet*,* so subservient. How do we hear that voice when that culture is so respectful?’ [ID129*,* Local Nurse Leader*,* Female].* It was considered that, to support retention, it was necessary for practices and other organisations to be more culturally aware and to consider that some activities which could be seen as helpful to collegiate working may have unintended consequences for those of differing backgrounds.

*We have a coffee break and everyone sits and talks. Even though that’s actually a nice thing*,* I noticed nursing associates*,* in particular*,* looking slightly uncomfortable in that situation…There was definitely a sense of them being more comfortable talking to me because I’m mixed race. I’m a bit in between because I’m a bit middle class*,* a bit working class*,* a bit white and a bit Caribbean. [ID 133, General Practice Nurse/trainee Advanced Nurse Practitioner and Local Medical Committee Lead Nurse, Female]*

### Ageism

Ageism was also considered by some to contribute to lack of recognition and value of the general practice nursing workforce. Employers were perceived to consider more experienced nurses as an expensive commodity who, in times of economic pressure, could be replaced by cheaper alternatives. Their knowledge was discounted in favour of lesser qualified and experienced team members. In this way, older nurses lacked the symbolic capital to maintain their economic position. Some participants reported feeling pushed out and unsupported in relation to caring responsibilities, or were made to think that age had negatively impacted on their performance, rather than an increasingly challenging workload, ‘*The workload has increased. At first I thought*,* “maybe I am slow*,* I’ve probably got menopause brain*,* I’m not as sharp”. You start doubting yourself…I bought a book on time management’ [ID 106*,* General Practice Nurse*,* Female].* Employers could behave in ways which made experienced nurses feel devalued and diminished, which ultimately impacted on retention.

*It was the checking up on things which was insulting. The straw that broke the camel’s back after the role that I’d been doing well. I’d not suddenly made errors that they felt I was no longer trustworthy. I’d begun to feel that [was] because of my age. It wasn’t because I wasn’t capable*. *[ID114, Retired General Practice Nurse, Female]*

Instead of actively working to retain experienced nurses, flexibility had decreased and there was a limited pipeline for recruitment of nurses in general practice. Instead of employers working to positively encourage retention, practices sometimes became less flexible and experienced older nurses felt pushed out.

*I just think was there a bit of ageism? I’ve spoken to a few other colleagues in other practices of a similar generation and they’re all saying a similar thing….The flexibility that there should be*,* and is talked about*,* often doesn’t exist. [ID 137: Retired Advanced Nurse Practitioner, General Practice Nurse and Fellowship Lead Nurse, Female]*

Furthermore, although this was a minority view, less experienced participants did not always value more experienced nurses. Previous negative experiences had led to discounting of more experienced colleagues and their cultural capital was diminished.

*[referring to legacy/mentoring roles] This is going to sound awful*,* but the last thing I would want as a student is to be paired up with a nurse who is just about to retire. I’ve worked with older nurses in general practice and in hospitals….they don’t like change. They don’t like students*,* new ways of thinking*,* of working. How can you be taught by somebody who isn’t willing to move with the times? I just find it bizarre. [ID 103 Nursing Associate, Female]*

## Discussion

In this paper we argue that the experiences of nurses working in general practice in relation to retention are underpinned by cultural and structural issues associated with the intersection of professional and societal level constructs of gender, age, race, ethnicity, medical hegemony, healthcare hierarchy, professional identity and social class which are highly complex and difficult to challenge. We suggest that these experiences fundamentally need to be viewed and addressed within a wider societal context to enable underlying long-term change to be achieved. In her work on progression in nursing, Williams [24] argues that societal issues such as gender, class, race and ethnicity intersect, and it is only by interrogating such issues at a conceptual level that greater understanding can be developed [24]. Our discussion unpicks these, and associated concepts, in relation to the retention of nurses in general practice using Bourdieu’s concept of Capital within which to situate findings.

Our findings indicated that gender played a significant role in the retention of nurses working in general practice. Nursing is traditionally seen as ‘pink collar’ work. It is highly gendered and poorly paid [35]. Only a small proportion of nurses working in general practice are male and specialities within healthcare where women predominate have been found to attract lower pay and less power and prestige [38]. While it is recognised that nursing more broadly remains stereotyped and undervalued because it is a predominantly female profession [39], our findings indicate that nurses working in general practice are dually affected. This is because the specific work is seen to be at the feminine and low status end of the nursing spectrum and involves what are often considered to be ‘softer skills’ of relationship development, negotiation and communication, rather than the ‘high tech’ more traditionally masculine end of the nursing spectrum [40]. What the nurses in our study valued so highly is often not seen as valuable by others.

Indeed, nursing roles considered to be closer to medicine, such as advanced nurse practitioners, were thought to be more highly valued in our study. Reynolds [41] points out that caring professions can be categorised as ‘caring about’ or ‘caring for’, with medicine classified as ‘caring about’, while nursing is seen to ‘care for’. ‘Caring about’ is considered caring at arm’s length [42, 43] and is attributed higher professional status [41] and consequently greater symbolic and economic capital than hands-on care [43]. Similarly, feminists argue that, for women, caring is seen as an innate part of their identity, rather than based on knowledge [44], and values culturally associated with women are ignored and demeaned [45]. Consequently, this confers little economic and symbolic capital with the socio-historic notion of nursing as a vocation and medicine as an elite profession [46] capitalising on this. Furthermore, direct patient care (caring for) has always been somewhat sidelined within the nursing hierarchy, with this aspect of care being delegated and associated with social class, race and gender and this dichotomy has persisted [23]. The role of advanced nurse practitioners in our study was seen to be closely aligned with the medical profession and focuses on more arm’s length ‘caring about’ practices such as prescribing and developing management plans, rather than hand’s on ‘doing’ work. This allowed advanced nurse practitioners to gain higher symbolic and economic capital than traditional nursing roles. However, this is only up to a point as, rather than levelling the hierarchy, it instead simply elongates with medicine remaining firmly at the top [46].

Linked to this are the difficulties articulated by participants in our study in negotiating appropriate terms and conditions and other aspects of their working lives, such as maternity pay, with their employers who they work closely with and who are also general practitioners. It has been argued that nursing continues to be dominated by a rigid healthcare hierarchy based on social status and gender differences [38]. The development of modern nursing and medicine was centred around a patriarchal model, with the subordination of working-class female nursing work underpinned by gender, class and power ideologies [47–49]. Consequently, development and control of the healthcare hierarchy was, and remains, successful from a medical perspective [49] in that medicine still benefits from being gendered male *as a profession* despite the increase in women in medicine, while nursing remains a distinctly female profession [36, 38]. This meant that some participants in our study were not able to effectively negotiate appropriate terms and conditions and consequently lacked economic capital. Unlike other professions, where remuneration improves when a skilled workforce is scarce, deprofessionalisation occurs where complex nursing work is devolved to nursing support roles. Nursing is vulnerable to deprofessionalisation, in part, because it fails to adequately protect its own professional boundaries and jurisdictions, while being positioned within a healthcare hierarchy where the established profession of medicine excludes other professions as a way of protecting its own privileges [50]. Nursing in general practice is in the invidious position of lacking the power to protect its own professional boundaries while experiencing exclusionary closure from medicine. In this way, lack of economic, cultural and social capital minimises the power of nurses working in general practice. Furthermore, as indicated by participants in our study, nurses in general practice feel that they are expected to negotiate with little support from professional associations, which also lack relative power, and within a hierarchy where their employers are also medics and are also more likely to be male [51]. While some study participants working at advanced clinical and leadership levels could be argued to have secured relatively healthy remuneration (≥ Band 7 equivalent), the ability of study participants to significantly change their economic circumstances [22], and the ability to achieve remuneration commensurate with their level of practice, remain limited by the perceptions and realities of the social and cultural capital of nursing and this has an ongoing negative impact on economic capital. Our assertion, drawn from the data, is that despite high levels of qualifications, skills and knowledge, and performing at an advanced level, nurses are fundamentally viewed within the context of societal and cultural perceptions of what nursing is and their economic capital is consequently limited.

The healthcare hierarchy, and associated power dynamics, are also implicated in the difficulty nurses working in general practice experience in getting a seat at the decision-making table and, even if they are there, having their voices heard and ideas implemented. Bassett [15] asserts that nurses in decision-making roles are required to overtly prove their credibility in order to be accepted, unlike their medical colleagues where higher status is assumed. Collaborative working has been found to be hindered by gendered status differentials which see women, and those whose work is considered to be *women’s work*, as less capable, competent and assertive. They are, therefore, less likely to have their voices heard [38]. Jackson [52] argues that nurses are socialised within a culture that requires subservience, compliance and ‘niceness’ which serve to silence nursing and maintain the status quo. Similarly, Stein [37] historically articulated the doctor-nurse game in which nurses follow ‘unwritten rules’ which conform to a traditional fixed healthcare hierarchy. Doctors remain at the top and severe penalties may be incurred for those who do not follow the rules of the game. While some, including Stein [53] himself argue that this game is no longer played, or that relationships are played out in different ways [54], it has more recently been established that the doctor-nurse game continues to be played out across different healthcare contexts [55–57] including general practice [46].

That nurses in our study felt it was difficult to contribute at a strategic level indicates that nursing in general practice can be seen to be hampered by the concepts of both the *glass ceiling* [58] and the *sticky floor* [59] with nurses dually affected by cultural and structural exclusion and the lack of opportunity provided by low status, poorly paid, low mobility, female-dominated occupations which lack support for career development and upward mobility. It is of note that in the more than two decades since the sticky floor was characterised [59] and Jackson’s more recent observations of subservient culture [52], that both report similar phenomena and little appears to have changed.

A recent systematic review and meta-analysis of barriers to advancement of female nurses in healthcare leadership [60], highlighted that female nurses lacked role models and formal and informal leadership training. They were also inhibited by social and cultural expectations of nursing as a female-gendered profession. This limited motivation of nurses to take on leadership roles. It was also found that medical dominance was perpetuated through hierarchical leadership structures. In addition, a recent scoping review of women’s leadership in healthcare [61] indicated that women were penalised for motherhood and faced lack of recognition, support and promotion chances. They also lacked affiliation to structural and cultural organisations such as ‘old boy’s networks’. In these ways, lack of economic, social and cultural capital are demonstrated. In our study, not only did general practices remain largely controlled by general practitioner partners and managers, other local organisations such as Integrated Care Boards and Primary Care Networks were also dominated by these groups with little recognition of the benefits of involving nurses at this level. Consequently, nurses working in general practice are undervalued and lack a voice [62]. Kalbarczyk et al. [61] argue that healthcare as a ‘business’ benefits from female leadership, resulting in improved outcomes. They found the most effective way of achieving this was through improving support and systems [61]. ‘Tokensim’, where women were minority members of the larger group, hindered women in leadership roles and contributed to maintaining majority organisational cultural privileges, with those in power having a vested interest in maintaining the hegemonic status quo [63]. In our study, nurses were very much a token minority at a decision-making level. The intersection between gender and social class meant that nurses were doubly disadvantaged within the healthcare hierarchy, and society more broadly, resulting in a lack of power to influence their work [22]. Not only does this relate to the historical development of nursing as a lower profession carried out by mainly working-class women [38], but it also highlights the lack of economic and symbolic capital the nursing profession continues to possess [22]. Relating these to our study, we argue that cultural and structural challenges in accessing leadership positions need addressing at a systems, organisational and societal level. Our call for cultural and structural change is reflected in studies of barriers to nursing more broadly [60] and wider healthcare leadership [61].

Snee and Goswarmi [22] utilise the concepts of economic, cultural and social capital to highlight that nursing is a relatively open profession in terms of social mobility *into* the profession (i.e. it is accessible to people, particularly women, of lower social class) and in our study, the availability of apprenticeships and support roles were considered to broaden access for people who would not otherwise be able to consider nursing as a career. However, unlike other professions, nursing is not a conduit to further upward social mobility, with the impact of original social class, and the associated lack of economic, cultural and social capital, enduring [22]. Consequently a ‘class ceiling’ where social mobility is stagnant and origin class advantage or disadvantage, in terms of economic, cultural and social capital, is retained [22]. In our study, it also appeared that some participants hit this *class ceiling*, where economic, social and cultural capital, both at a societal level, and within the healthcare hierarchy, prevented their worth and contribution being recognised, and their voice being heard. Consequently, it is not simply enough to gain a seat at the decision-making table, but fundamental structural and cultural issues need to be addressed if nurses are to be valued and retention improved.

In our study, the intersection of gender and social class was further complicated when interacting with other aspects of identity such as race, ethnicity or age in terms of retention. Indeed, internal nursing hierarchies based on the intersection of gender, race, ethnicity and class have consistently resulted in those with multiple disadvantage being seen as *less than* and employed in less prestigious, lower wage employment and specialities, which reinforces disadvantage and exploitation [23]. In this way, lack of economic capital (to command adequate terms and conditions or take one’s labour elsewhere) and symbolic capital (historically carried over from colonialism and empire) perpetuate divisive patterns of disadvantage [23].

People working in the National Health Service from global majority backgrounds and international recruits are more likely to be affected by predictors of attrition such as poor pay and career progression and to suffer from racial harassment [64], as well as being more likely to face disciplinary action [65]. The impact of racism is multifactorial and includes economic, emotional, ethical, legislative and patient care implications [65]. Although there is a recognised gender advantage for men in nursing, in terms of career progression, this has been found to be negated by exclusion and marginalization based on race and ethnicity [24]. While, for those who are female and from minoritised groups, lack of structural and cultural support is exacerbated [63]. However, evidence exploring and understanding why staff from diverse ethnic groups may be more or less likely to stay in employment is lacking, as is awareness of what encourages retention and how well current retention strategies work [64]. It is important, then, that these issues are addressed with a specific focus on the needs of those working in general practice.

Ageism has significant consequences for individuals, the profession, and healthcare systems more broadly as it impacts on emotional well-being, performance and retention [66]. In our study, ageism impacted on retention in a number of ways and was underpinned by symbolic and economic capital. Participants experienced discounting and diminishing from employers and sometimes from younger nurses. This was reflected in recent systematic reviews [25, 66] where both younger nurses and managers failed to recognise older nurses’ value (cultural capital) and perceived them to be slower and less able than younger nurses. As in our study, it has previously been found that older nurses perceived managers were seeking to replace them with cheaper alternatives [67] negatively impacting economic capital. Additionally, older nurses often felt they were excluded from decision-making to a greater extent than their younger nursing colleagues [68] highlighting the reduced symbolic capital held by older nurses. Given that nursing in general practice is an ageing workforce with a depth and breadth of skill, knowledge and experience, it is necessary for employers, and wider healthcare organisations, to support more experienced nurses to remain in general practice and to avail themselves of this experience at a strategic and decision-making level.

Participants in our study highlighted a number of factors contributing to attrition and retention in nursing in general practice that were underpinned by the intersection of gender, social class, ageism, race, ethnicity, medical and managerial hegemony and professional identity. These issues are not unique to this context, but have been identified in other aspects of nursing, including executive leadership [15, 16] where gender, power, hegemonic dynamics and assumptions made about nursing and nurses coalesce to disadvantageously position nurses. We argue, then, that while our study explores retention of a specific group within a particular working environment, our findings speak to a wider issue experienced by the nursing profession, that of the professional discounting of nurses *because they are nurses* [15]. Using Bourdieu’s concept of Capital [20, 21], we argue that nursing as a profession lacks the cultural, economic and social capital within which to negotiate within the wider healthcare network and there are specific challenges for nurses working in general practice. These impact on their position within general practice, and ultimately on retention. In the first instance it is important to raise these issues to a conscious level. Organisational and cultural strategies have the potential to support nurses to negotiate their professional pathway [66]. However, there is a dearth of such initiatives and employers and policy makers should be encouraged to develop strategies to address specific issues such as gender [61], ageism [66], social class [22], race [65] and ethnicity [64]. Nevertheless, until the gendered and class position of nursing, and its relationship within the healthcare hierarchy, professional identity and medical hegemony, as well as other factors such as age, race and ethnicity, are addressed at professional, cultural, structural and societal levels, the extent to which any initiatives to address retention are likely to be successful remain unclear.

### Strengths and limitations

Strengths of the study include the depth of data generated and successful targeted recruitment for maximum variation. This enabled a deep dive into understanding of experiences of nurses working in general practice and the underpinning cultural and structural issues associated with retention. A potential limitation of social media recruitment is sample homogeneity [69]. We addressed this by phasing recruitment and utilising a demographic questionnaire to assess representation, while also recruiting simultaneously via broader professional networks. Through this we were able to recruit for maximum variation in relation to role, gender, practice demographics and geographical location. Age and ethnicity mirrored national rates, although the number of participants from global majority backgrounds was small and findings need to be viewed within this context. Further research is necessary to explore the experiences of this group in greater detail. We were unsuccessful in recruiting from the south west of England. As this is one of a number of areas impacted by deprivation and rural isolation, this is a limitation.

A further potential limitation was a lack of input from other healthcare professionals, managers, employers and decision-makers beyond nursing. Engaging with patients and carers might also have provided differing viewpoints. However, practical decisions were made due to limited funding and timeframe for the study. Additionally, expanding out from a largely nurse-centred sample may have detracted from the experiences of nurses themselves.

By drawing on the principle of naturalistic generalisability, we propose that the shared nature of the experiences of nurses in our study, and the organisational context highlighted, may be recognisable to others. However, we do not wish to overstate inferences from interview data and its use in inform future policy and practice should be viewed within this context.

## Conclusions

The World Health Organisation [70] argues for the development of evidence-based nursing retention strategies that are tailored to local context and tackle inequalities. The intersection of underpinning explanatory cultural and structural factors associated with social class, age, gender, race, ethnicity, medical hegemony, healthcare hierarchy and professional identity highlighted in this paper indicate why some retention issues in general practice are so difficult to resolve and continue to endure. We argue that the first steps are to recognise these factors and raise them to a conscious level. We suggest that, unless these fundamental issues are acknowledged and work undertaken to address them, strategies to meet the needs of nurses working in general practice, and potentially nursing retention more broadly, may be unsuccessful.

## Supplementary Information

Additional file 1. BMC HSR GenRet Study Participant Demographic Template.dox

Additional file 2. BMC HSR Topic Guide.dox

Additional file 3. GenRet BMC Additional Tables.docx

## Data Availability

The data sets generated and/or analysed during the current study are not publicly available due the being an in-depth qualitative data set for a specific group of participants who could potentially be identifiable if whole transcripts were publicly available. Data sets are available from the corresponding author on reasonable request.
