# Supplementary material for: “I’m a bit middle class, a bit working class, a bit white and a bit Caribbean” - the retention of nurses in general practice and the intersection of professional and societal level cultural and structural issues: a qualitative interview study
Source: BMC Health Serv Res. 2025 Oct 9;25:1339. doi: 10.1186/s12913-025-13420-2 (PMC12512638; doi:10.1186/s12913-025-13420-2)
Supplement: Supplementary file 1 — Additional file 1. BMC HSR GenRet Study Participant Demographic Template.dox [file 12913_2025_13420_MOESM1_ESM.docx]

**Exploring the relationship between cultural and structural workforce issues and the retention of nurses working in general practice: A qualitative study**

Demographic information

*Please complete as much applicable information as you can. This will help us to make sure we have recruited a variety of participants from a range of different general practices and experiences. Thank you.*

| **ID** |  |
| --- | --- |
| **Job title** |  |
| **NMC PIN** |  |
| **Brief description of role** |  |
| **Qualifications** |  |
| **Years qualified** |  |
| **Age range:**  20-29  30-39  40-49  50-59  60-70  70+ |  |
| **Gender** |  |
| **Ethnicity** |  |
| **Approximate pay or Agenda for Change banding equivalent** |  |
| **Size/description of nursing team** |  |
| **Practice description:**   - Size of practice - Part of country - Rural/urban/inner city - Practice Postcode |  |
| **Personal email address to send voucher** |  |

*To be completed by researcher:*

| **National General Practice Profile data** ([**https://fingertips.phe.org.uk/profile/general-practice**](https://fingertips.phe.org.uk/profile/general-practice)**) or** [**https://statswales.gov.wales/Catalogue/Health-and-Social-Care/General-Medical-Services/General-practice-population**](https://statswales.gov.wales/Catalogue/Health-and-Social-Care/General-Medical-Services/General-practice-population) | |
| --- | --- |
| Number of patients registered |  |
| Life expectancy |  |
| Deprivation score |  |
| Ethnicity estimate |  |
| CQC rating |  |

| **Date recruited** |  |
| --- | --- |
| **Consent obtained** |  |
| **Interview arranged** |  |
| **Interview completed** |  |
| **Recording sent for transcription** |  |
| **Transcription completed** |  |
