# Supplementary material for: “I’m a bit middle class, a bit working class, a bit white and a bit Caribbean” - the retention of nurses in general practice and the intersection of professional and societal level cultural and structural issues: a qualitative interview study
Source: BMC Health Serv Res. 2025 Oct 9;25:1339. doi: 10.1186/s12913-025-13420-2 (PMC12512638; doi:10.1186/s12913-025-13420-2)
Supplement: Supplementary file 2 — Additional file 2. BMC HSR Topic Guide.dox [file 12913_2025_13420_MOESM2_ESM.docx]

**
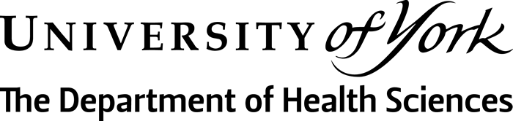
**

**GenRet Study**

**Exploring the relationship between cultural and structural workforce issues and the retention of nurses working in general practice: A qualitative study**

**Interview Guide for GenRet**

This topic guide summarises the main areas to be explored in each interview. As with any qualitative interviews, these headings and questions are intended as a starting point to ensure the primary issues are covered, whilst allowing flexibility for new issues to be explored. It is not anticipated that all interviewees will be asked about all topics, or that the interview will follow a question-and-answer format. The researcher will ask the participant to elaborate on any responses where appropriate. Preliminary analysis of data from earlier interviews will shape the topics covered in later interviews.

**GenRet Study Specific Topic Guide:**

We would like to find out about your experiences of working in general practice and your thoughts about issues around retention of nurses working in general practice.

1. Background information

- Role and profession/background
- How long in profession?
- How long in general practice?
- Worked in other practices/roles previously?

1. What influenced you/helped you decide to work in general practice?

1. Did you have any experience of general practice before you started working there?

- Previous roles in general practice (e.g. admin. HCA)?
- Experience as a nursing student?

1. Is working in general practice what you expected?
2. What are the main reasons you work in general practice?
3. Could you describe a typical working day?
4. What parts of your work do you most enjoy and which do you find more difficult?
5. Do you plan to continue working in general practice?

- Why/not?
- What has influenced this decision?
- Have things changed post Covid?
- Do your nursing colleagues plan to continue to work in general practice?
  1. What do you think are the challenges in retaining nurses in general practice?
  2. Are there any specific issues which you think make nurses choose to leave general practice?
  3. Are there any structural issues that impact on retention?

For example:

- pay and conditions
- pension
- organisational issues
- involvement in decision-making
- workload

12. Are there any issues which could be described as workplace culture issues which might impact on retention?

For example:

- nurses’ role in governance at practice level (& relation to ARRS)
- support
- hierarchy
- loyalty to practice
- culture of overwork/presenteeism
- interprofessional tensions
- managerial tensions
- Do any issues within nursing teams/ relationships affect retention?
- Are there any issues related to inclusivity, diversity and equality which may impact to retention?
  1. Are there any external factors which may influence retention?

For example:

- Support from professional associations
- Family/caring responsibilities
- External commitments
- Media/public perceptions

             Follow up/prompt: Do you think nurses in general practice are valued?

- by their employers?
- by GPs?
- other colleagues?
- by patients?

- 1. Do you have a role of educating/supervising others in general practice and does this affect retention?

e.g.

- UG nurses/NAs/HCAs
- medical students
- PG RNs Drs
- ARRS roles
  1. What do you think would support retention?
- What would make you want to stay in general practice?
- Is this achievable in general practice as it currently stands?
  1. Has your practice/PCN taken any steps to retain nurses? Have nurses themselves had any input or influence on this?

e.g.

- flexible working
- legacy roles (supporting/training nurses new to GP)
- addressing disparity of T&Cs?

- 1. Would you recommend a career in general practice to other nurses?
- Why/not?

- 1. Where do you see nursing in general practice in 10 years’ time? Do you think it is sustainable in its current form?

- 1. Is there anything you would like to add that you think is important or that has been missed?

**End of Interview**
